# Spectrum effect correlation of yangyin tongnao granules on cerebral ischemia-reperfusion injury rats

**DOI:** 10.3389/fphar.2022.947978

**Published:** 2022-08-09

**Authors:** Yangyang Zhang, Li Yu, Jiehong Yang, Zhishan Ding, Yu He, Haitong Wan

**Affiliations:** ^1^ School of Basic Medical Sciences, Zhejiang Chinese Medical University, Hangzhou, China; ^2^ School of Life Sciences, Zhejiang Chinese Medical University, Hangzhou, China; ^3^ School of Medical Technology and Information Engineering, Zhejiang Chinese Medical University, Hangzhou, China; ^4^ School of Pharmaceutical Sciences, Zhejiang Chinese Medical University, Hangzhou, China

**Keywords:** yangyin tongnao granules, cerebral ischemia-reperfusion injury, HPLC fingerprint, mathematical model, spectrum-effect relationship

## Abstract

Yangyin Tongnao Granules (YYTNG), as traditional Chinese medicine (TCM) compound preparation, have a good curative effect on cerebral ischemia-reperfusion injury. This study aimed to investigate the relationship between the active components of YYTNG in the plasma and the inflammatory response in cerebral ischemia-reperfusion injury rats. High-performance liquid chromatography (HPLC) was conducted to determine the fingerprints at different time points of middle cerebral artery occlusion (MCAO) rats after the administration of YYTNG at different times points. Enzyme-linked immunosorbent assay (ELISA) was performed to detect the levels of interleukin-18 (IL-18) and tumor necrosis factor-α (TNF-α) in the plasma of MCAO rats at different time points. The spectral-effect relationship between the YYTNG fingerprints and inflammatory indexes *in vivo* was established by combining three different mathematical models, grey correlation, multiple linear regression, and partial least-square method. The results revealed that each chromatographic peak in the HPLC of the plasma exhibited a certain correlation with the inflammatory index, in the following order: *P2* >*P6* >*P5* >*P1* >*P3* >*P4*. Therefore, this study successfully established the spectrum-effect correlation of YYTNG on cerebral ischemia-reperfusion injury rats. The results provide a certain guiding ideology for the analyses of the relationship between fingerprints and the pharmacodynamics of TCM prescriptions.

## 1 Introduction

Since ancient times, stroke has been regarded as critical and severe disease, with ischemic stroke being more common than hemorrhagic stroke. The pathological process of ischemic stroke is quite complex, and its pathogenesis involves energy metabolism disorder, free radical injury, excitatory amino acid toxicity, and inflammatory reaction (Wang and Ma, 2018). As one of the common diseases of the nervous system, ischemic stroke has been associated with high disability and high mortality rates, which causes immense pain to patients and their families.

Several studies on the mechanism of brain injury after ischemia have focused on the inflammatory process after ischemia-reperfusion (Liu et al., 2019; Shi et al., 2019; Liu et al., 2020; Mo et al., 2020). It has been proven that a strong inflammatory response after cerebral ischemia is the causative factor for secondary injury of brain cells (Jayaraj et al., 2019; Seiya et al., 2020). The expression of various inflammatory mediators increases significantly during cerebral ischemia, which is responsible for the dual effects of brain injury and brain protection (Poinsatte et al., 2015).

Yangyin Tongnao granules (YYTNG) are a modern compound preparation of traditional Chinese medicine (TCM). YYTNG is composed of *Rehmannia glutinosa* (Gaertn.) DC [Orobanchaceae], *Pueraria montana var. Lobata* (Willd.) Maesen and S.M.Almeida ex Sanjappa and Predeep [Fabaceae], *Astragalus mongholicus* Bunge [Fabaceae], *Conioselinum anthriscoides ‘Chuanxiong’* [Apiaceae], *Dendrobium nobile* Lindl [Orchidaceae]*,* and Hirudo in the ratio of 1:1.2:1:0.67:1:0.2 (Wang et al., 2019). It has also been approved for clinical research (approval number: 2003L00206), and it has completed phase II and phase III clinical trials. According to the TCM theory, YYTNG nourishes Yin and replenishes qi (Wan et al., 2015). In Western medicine, it has been demonstrated to promote blood circulation and improve microcirculation, especially in the treatment of cardiovascular and cerebrovascular diseases (Liang and Su, 2015). Previous studies have shown that YYTNG exerts antioxidant anti-inflammatory effects, inhibits neuronal apoptosis, and improves blood system diseases and other functions (Rong et al., 2017; Rong et al., 2018). The relationship between the spectrum and the effect is often employed to evaluate the potential correlation between the active ingredients of TCM and drug action (Jidan et al., 2019; Zhou et al., 2019). The study of the relationship between the spectrum and the effect has established a representative method to identify the main active ingredients related to the pharmacological effects of the complex TCM formula. Presently, the data processing of the relationship between spectrum and effect mainly includes correlation analysis (Zhou et al., 2021), clustering analysis (Gao et al., 2019), and regression analysis (Liu et al., 2019).

In this study, inflammatory factors (such as tumor necrosis factor-α [TNF-α] and interleukin-18 [IL-18]) were selected to establish the relationship between the high-performance liquid chromatography (HPLC) fingerprint of YYTNG in the middle cerebral artery occlusion (MCAO) rats and inflammatory indexes.

## 2 Materials and methods

### 2.1 Experiment animals

Six male Sprague Dawley (SD) rats (weight: 280–300 g; SPF) were provided and raised by the Institutional Animal Care and Use Committee at the Zhejiang Chinese Medical University. The license number of the experimental animals was SYXK (Zhejiang) 2018-0012.

### 2.2 Chemicals and reagents

YYTNG (product batch number: 170322) was manufactured by Shanxi Buchang Pharmaceutical Co., Ltd. China. Heparin Sodium (product batch number: 1170GR005) was purchased from Shanghai McLin Biochemical Technology Co., Ltd.

Methanol (product batch number: 20105218) and acetonitrile (product batch number: LI70T97) of chromatographic grade were obtained from the Tedia Company. Formic acid (product batch number: 20180709) was provided by Shanghai Aladdin Biochemical Technology Co., Ltd. TNF-α (product batch number: MB-1721A) and IL-18 (product batch number: MB-1735) ELISA kits were purchased from the Jiangsu Enzyme Label Biotechnology Co., Ltd.

### 2.3 Quality control of yangyin tongnao granules

The preparation technology of YYTNG was based on the existing relevant report of our research group (Wang et al., 2019). Meanwhile, the fingerprints and ingredients of determination were conducted by our research group (Wang et al., 2019; Yu et al., 2021). YYTNG is composed of puerarin, calycosin, formononetin, ferulic acid, daidzein, and other ingredients.

### 2.4 Instruments and chromatographic conditions

The analysis was performed on the Agilent 1200 Series System (Agilent Technologies, United States ) equipped with a diode array detector. Chromatographic separation was achieved on the Eclipse XDB-C_18_ (4.6 × 250 mm, 5 µm) analytical column at 25°C. A gradient elution program was conducted for chromatographic separation with the mobile phase acetonitrile (A) and 0.5% formic acid aqueous solution (B) as follows: 0–10 min (A: 2%–10%), 10–29 min (A: 10%–12%), 29–35 min (A: 12%–15%), 35–50 min (A: 15%–25%), 50–65 min (A: 25%–60%), 65–70 min (A: 60%–90%), 70–80 min (A: 90%–2%), 80–85 min (A: 2%). The flow rate was set to 1.0 ml/min. Then, 10 μL of the sample was injected automatically.

### 2.5 Middle cerebral artery occlusion models establishment

The rats were anesthetized via intraperitoneal injection with 3% (w/v) sodium pentobarbital (45 mg/kg). The MCAO model was established according to the modified Zea Longa method (Longa et al., 1989; Yu et al., 2018). Briefly, the right common carotid artery (CCA), internal carotid artery (ICA), and external carotid artery (ECA) were separated. A small cut was made on the CCA with ophthalmic scissors. Nylon thread was gently pushed into the ICA from this small cut on the CCA. The black mark of the nylon thread stopped when it reached the trigeminal orifice. After 1 h of ischemia, the nylon thread was pulled out and the incision was sutured before reperfusion. The rats with Horner’s syndrome in the right eye, long and short legs in the left forelimb when lifting the tail, and hemiplegic behavior when walking freely were selected for the subsequent analyses.

### 2.6 Reagent preparation and dosage

Heparin sodium powder (0.1722 g) was precisely weighed and added to a centrifuge tube, to which 4 ml of normal saline was added. The heparin sodium powder was then fully dissolved via ultrasound. The resultant heparin sodium solution was stored in a refrigerator at 4°C until further use.

After MCAO modeling, the rats were administered with YYTNG orally 1 h after reperfusion. Based on the clinical conversion dose, adult dose/60 kg × 6.3 (adult dose: 16.5 g), the drug dose for a rat was determined to be 3 times that of the adult dose, that is, 5.20 g/kg.

### 2.7 Preparation of drug-containing plasma

The blood samples were collected from the mandibular vein of MCAO/R rats at 2, 4, 6, 8, 12, 24, 48, and 72 h after the first dose (immediately administered with the same amount of YYTNG as the first dose after the blood sampling at 24 and 48 h). The plasma was placed in a centrifuge tube containing 20 µL of the heparin sodium solution, shaken up and down gently, and then fully mixed. Then, the mixture was placed at room temperature for 30 min and centrifuged at 4,500 rpm for 15 min. The supernatant was used as the medicated plasma. The samples were placed in the refrigerator at −20°C overnight, and then transferred to a refrigerator at −80°C for storage until further use.

### 2.8 Sample pretreatment

The medicated plasma (100 µL) was precisely aspirated, to which 3 times the amount of methanol was added to remove the protein, and centrifuged at 12,000 rpm for 12 min at 4°C. Then, the supernatant was aspirated, and subjected to nitrogen blowing for concentration and enrichment. Finally, 100 µL of 50% methanol was added to reconstitute the sample.

### 2.9 Preparation of the blank plasma

Normal SD rats were fasted (no water deprivation) for 12 h, and their blood was sampled and processed according to the aforementioned sample pretreatment methods to obtain the corresponding blank plasma.

### 2.10 Establishment of fingerprints of yangyin tongnao granules at different time points in middle cerebral artery occlusion rats

The reconstituted plasma samples were centrifuged at the speed of 12,000 rpm at 4°C for 12 min, followed by aspiration of the supernatant for HPLC detection. The plasmas of each rat at different time points were analyzed by the established HPLC method. The HPLC charts of each time point were subsequently obtained. After importing the HPLC charts at each timepoint into the Chinese medicine chromatographic fingerprint similarity evaluation system (version 2012A), the fingerprint of the rat medicated plasma was automatically generated.

### 2.11 Detection of the inflammatory indexes in middle cerebral artery occlusion rats

We took appropriate amounts of the medicated plasma at different time points and detected the changes in the levels of inflammatory factors (i.e., IL-18 and TNF-α) in accordance with the instructions of the ELISA kit. Then, the microplate reader was used to determine the OD value at the wavelength of 450 nm.

### 2.12 Evaluation of the spectrum-effect relationship between the fingerprint of yangyin tongnao granules and inflammatory indexes of cerebral ischemia-reperfusion injury

#### 2.12.1 Data normalization processing for inflammation indicators

Both IL-18 and TNF-α were indicators of inflammation, they shared some inherent correlation. Accordingly, the entropy weight method was applied to weigh these two indicators, after which they were normalized to obtain the comprehensive evaluation value of the drug effect. The calculation of the entropy weight method was performed using the following steps:

1) Data standardization

$$r_{ij}=\frac{x_{ij}-\text{min}\left(x_{j}\right)}{\max\left(x_{j}\right)-\text{min}\left(x_{j}\right)}$$ (1)

$$r_{ij}=\frac{\max\left(x_{j}\right)-x_{ij}}{\max\left(x_{j}\right)-\text{min}\left(x_{j}\right)}$$ (2)

where Eq. 1 presents a positive data standardization, while Eq. 2 presents a negative data standardization. $\text{min}\left(x_{j}\right)$ is the minimum value of column *j*th data; $\text{max}\left(x_{j}\right)$ is the maximum value of column *j*th data; $\text{min}\left(x_{j}\right)$ is the minimum value of column *j*th data; and $\text{max}\left(x_{j}\right)$ is the maximum value of column *j*th data.

2) Calculation of the information entropy *E*_*j*_ of each index

$$E_{j}=-\frac{1}{\text{ln}\left(n\right)}\times \overset{n}{\underset{i=1}{\sum }}p_{ij}lnp_{ij}$$ (3)

where, $p_{ij}=r_{ij}/\overset{n}{\underset{i=1}{\sum }}r_{ij}$ , and *n* is the number of experiments.

3) Determination of the weight of each indicator

$$W_{j}=\frac{1-E_{j}}{k-\sum_{j=1}^{k}E_{j}}$$ (4)

where *k* is the number of evaluation indexes.

#### 2.12.2 Grey correlation analysis

Grey correlation analysis (Sarraf and Nejad, 2020) refers to the method of applying a grey correlation degree to analyze the grey systems with partly known and partly unknown information. Most of the chromatographic peaks in the fingerprint are unknown compounds, and the efficacy of these compounds in animals is unknown. Therefore, the area of six common peaks was used as the independent variable, and the values of pharmacodynamic comprehensive evaluation acted as a dependent variable for grey correlation analyses. The gray correlation analysis steps are described below:

1) The establishment of the original matrix $x_{i}$ of the related indicators

$$x_{i}=x_{i}\left(1\right),x_{i}\left(2\right),\cdots x_{i}\left(k\right),$$

where $x_{i}\left(k\right)$ represents the original data of the *i*th peak at the *k*th time point.

2) Using the average value $x_{i}\left(1\right)$ of each peak area at each time as the mother sequence, the solution of the initial value transformation matrix $x_{i}^{\prime }$

$$x_{i}^{\prime }=\left(x_{i}\left(1\right)/x_{i}\left(1\right),x_{i}\left(2\right)/x_{i}\left(1\right),\cdots x_{i}\left(k\right)/x_{i}\left(1\right),\right)=\left(x_{i}^{\prime }\left(1\right),x_{i}^{\prime }\left(2\right),\cdots x_{i}^{\prime }\left(k\right)\right)$$ (5)

3) Difference sequence $\Delta_{0i}\left(k\right)$

$$\Delta_{0i}\left(k\right)=\left|x_{0}^{i}\left(k\right)-x_{i}^{\prime }\left(k\right)\right|=\left(\Delta_{0i}\left(1\right),\Delta_{0i}\left(2\right),\cdots \Delta_{0i}\left(k\right)\right)$$ (6)

4) Calculation of the correlation coefficient $\delta_{0i}\left(k\right)$

$$\delta_{0i}\left(k\right)=\frac{\min\min\Delta_{0i}\left(k\right)+\varphi \times \max\max\Delta_{0i}\left(k\right)}{\Delta_{0i}\left(k\right)+\varphi \times \max\max\Delta_{0i}\left(k\right)}$$ (7)

where, φ is the resolution coefficient, which can increase the significance of the difference between the correlation coefficient $\delta_{0i}\left(k\right)$ , φ∈(0,1) , generally 0.5.

5) Calculation of the correlation degree $r_{i}$

$$r_{i}=\frac{1}{n-1}\overset{n}{\underset{k=1}{\sum }}\delta_{i}\left(k\right)$$ (8)

The more the $r_{i}$ value tends to 1, the better the correlation between the two. $r_{i}$ > 0.6, which indicates a certain correlation between the chemical components represented by the chromatographic peak and the values of pharmacodynamic comprehensive evaluation. $r_{i}$ > 0.8 indicates a better correlation between the chemical components represented by the chromatographic peak and the values of pharmacodynamic comprehensive evaluation. $r_{i}$ > 0.9, indicates an excellent correlation between the chemical components represented by the chromatographic peak and the values of pharmacodynamic comprehensive evaluation.

#### 2.12.3 Multiple linear regression

The analysis of the fingerprints of YYTNG in MCAO rats demonstrated six common peaks in the HPLC chart of the medicated plasma at different time points. Moreover, the structure and name of the chemical ingredients represented by these six chromatographic peaks are unknown. Meanwhile, the relationship between the ingredients and the efficacy was relatively vague. Therefore, the data were subjected to a correlation analysis using the calculation formula shown in Eq. 9:

$$r\left(X,Y\right)=\frac{Cov\left(X,Y\right)}{\sqrt{Var\left(X\right)\times Var\left(Y\right)}}$$ (9)

#### 2.12.4 Partial least-square method

The partial least-square method is a mathematical optimization technique that combines multiple linear regression analysis, canonical correlation analysis, and principal component analysis (Yu, 2014). This method optimizes the best matching function between the independent and dependent variables by minimizing the error sum of squares. The calculation step of the partial least-square method is depicted below:

1) Data standardization

The obtained peak area and the normalized comprehensive score were standardized according to Eqs.1, 2

2) The principal component extraction of the two variable groups was performed to maximize the correlation

The corresponding formula is depicted in Eqs 10, 11.

$$t_{1}=w_{11}x_{1}+w_{12}x_{2}+\cdots +w_{1m}x_{m}$$ (10)

$$\;u_{1}=v_{11}y_{1}+v_{12}y_{2}+\cdots +v_{1p}y_{p}$$ (11)

where $t_{1}$ is the first principal component of the independent variable, and $u_{1}$ is the first principal component of a dependent variable.

3) The establishment of regression between $y_{1},y_{2},\cdots ,y_{p}$ and $t_{1}$

$$y_{p}=r_{1p}t_{1}+r_{2p}t_{2}+\cdots +r_{kp}t_{k}$$ (12)

where *k* is the number of principal components extracted from the independent variable.

4) The establishment of a partial least-squares regression equation between the independent variable (*x*) and the dependent variable (y)

$$y_{p}=r_{1p}\left(w_{11}x_{1}+w_{12}x_{2}+\cdots +w_{1m}x_{m}\right)+r_{2p}\left(w_{21}x_{1}+w_{22}x_{2}+\cdots +w_{2m}x_{m}\right)+\cdots +r_{kp}\left(w_{k1}x_{1}+w_{k2}x_{2}+\cdots +w_{km}x_{m}\right)$$ (13)

The functional relationship between the peak area of six common peaks and the values of the pharmacodynamic comprehensive evaluation was established through the partial least square analysis method using the MATLAB R2018 b software.

## 3 Results

### 3.1 High-performance liquid chromatography fingerprints of yangyin tongnao granules in rats with cerebral ischemia-reperfusion

**FIGURE 1:**
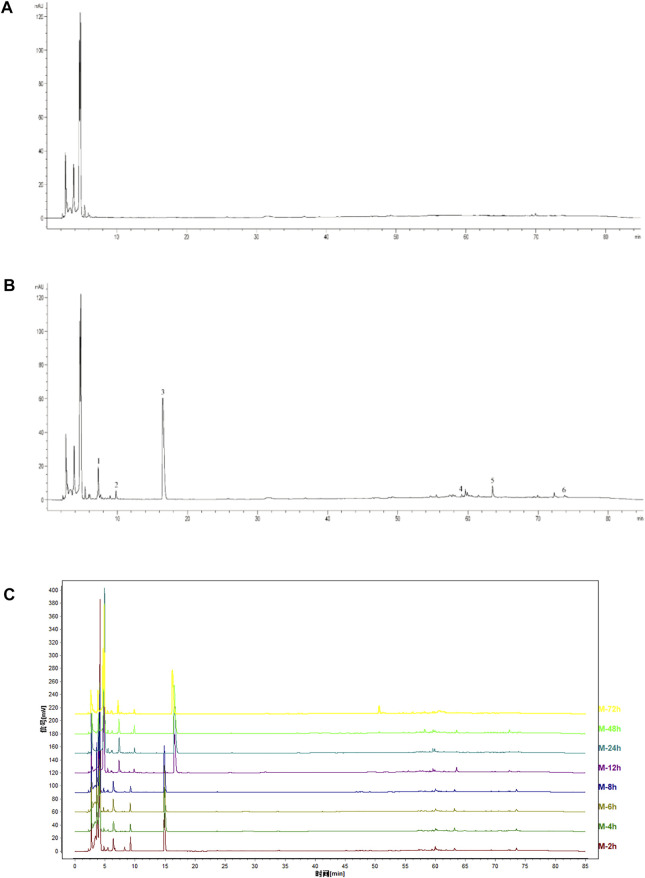
HPLC chromatograms of YYTNG in MCAO rats. **(A)** Blank plasma; **(B)** Medicated plasma of 2 h after administration; **(C)** Fingerprints at different time points.

The HPLC fingerprint of YYTNG in rats with cerebral ischemia reperfusion demonstrated that the blank plasma of the rat was shown in Figure 1A. The six common peaks occurred in the medicated plasma of rats, represented as 1-6 in Figure 1B. The fingerprints of medicated plasma of rats at different time points are shown in Figure 1C. The area of each peak in the medicated plasma at different time points is shown in Table 1. The six common peaks were represented as P1-P6.

**TABLE 1:** The peak areas in the rat plasma at different time points ( $\bar{x}$ ±s, *n* = 6).

	2 h	4 h	6 h	8 h	12 h	24 h	48 h	72 h
*P1*	181.5 ± 57	184.1 ± 30.2	171.8 ± 18.2	172.1 ± 18.2	141.2 ± 20.4	183.8 ± 47.3	176 ± 22.4	159.9 ± 33
*P2*	107.1 ± 23	82.1 ± 3.3	85.6 ± 9.5	59.4 ± 9.5	41.5 ± 2.5	48.8 ± 7.8	94.2 ± 3	53.7 ± 6.1
*P3*	921.8 ± 293.4	1050.2 ± 173.2	907.6 ± 38.2	892.8 ± 38.2	949.5 ± 103.3	1266.5 ± 297.6	1158.8 ± 142	1003.1 ± 83.3
*P4*	12.3 ± 1.4	11.6 ± 1.5	11.2 ± 0.9	28.4 ± 0.9	15.7 ± 4.6	21.1 ± 6.3	33.3 ± 13.5	45.7 ± 23
*P5*	36 ± 5.4	40.1 ± 9.6	33.5 ± 5.5	86.9 ± 5.5	66.7 ± 0.1	28.6 ± 24	57.1 ± 36.7	72.6 ± 49.4
*P6*	27.7 ± 7.4	31 ± 1.6	31.3 ± 1.6	18.7 ± 1.6	12.2 ± 0.2	16.3 ± 3.7	15.4 ± 1.4	13.6 ± 1.1

### 3.2 Effect of yangyin tongnao granules on inflammatory factors at different time points in rats with cerebral ischemia-reperfusion

**FIGURE 2:**
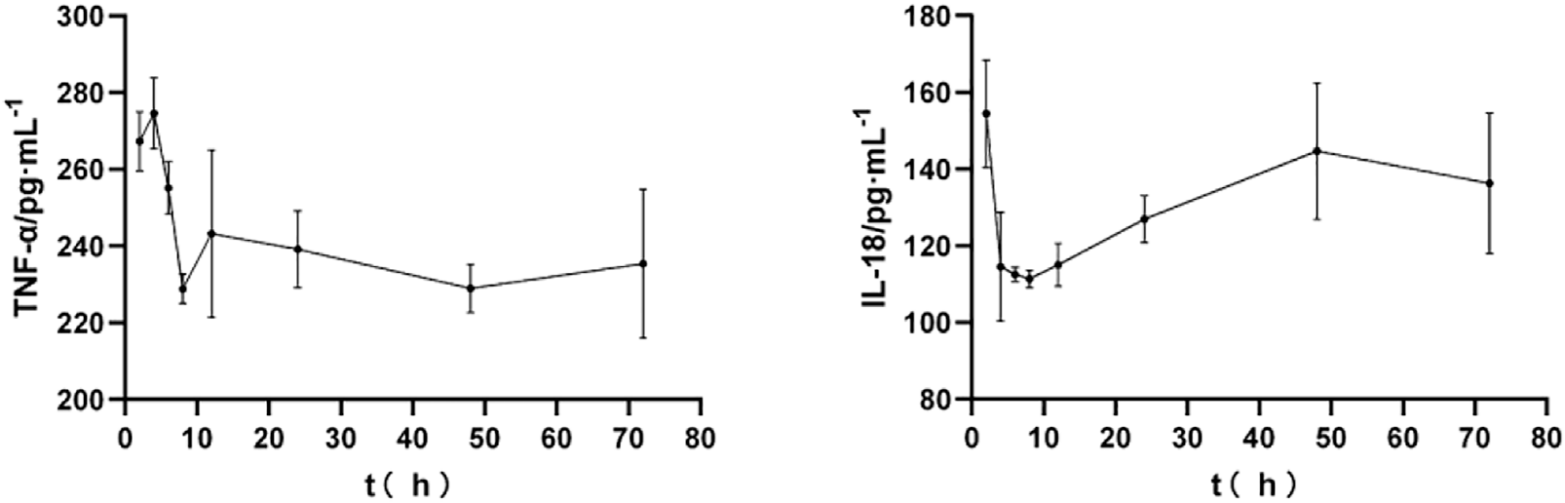
Effect of YYTNG on the inflammatory factors at different time points in MCAO rats ( $\bar{x}$ ±s, *n* = 6)

As shown in Figure 2, after the SD rats were modeled by MCAO and orally administered YYTNG, the levels of TNF-α and IL-18 in the plasma of the rats with cerebral ischemia-reperfusion at different time points revealed a downward trend. The levels of TNF-α and IL-18 reached the lowest point at 8 h, followed by a gradual increase.

### 3.3 Analysis of the results of spectrum-effect correlation

#### 3.3.1 The results of normalization of inflammatory indexes

The entropy weight method was applied to assign values to each inflammation indicator. First, all raw data were standardized according to Eqs 1, 2. Second, according to Eq. 3, the information entropies IL-18 and TNF-α were 0.896 and 0.937, respectively. Finally, according to Eq. 4, the calculated weights of IL-18 and TNF-α were 0.621 and 0.379, respectively. Therefore, the formula used to obtain the pharmacodynamic comprehensive evaluation values was: *Y* = 0.621 *× Y1* + 0.379 × *Y2*, where, *Y* represented the values of a pharmacodynamic comprehensive evaluation, *Y1* represented the level of IL-18 inflammatory factor, and *Y2* represented the level of TNF-α inflammatory factor.

The peak areas and pharmacodynamic comprehensive evaluation values (*Y*) of the six common peaks (*P1*-*P6*) at different time points were obtained. The results are depicted in Table 2.

**TABLE 2:** Peak areas and pharmacodynamic comprehensive evaluation value at different time points ( $\bar{x}$ ±s, *n* = 6).

	2 h	4 h	6 h	8 h	12 h	24 h	48 h	72 h
*P1*	181.5	184.1	171.8	172.1	141.2	183.8	176	159.9
*P2*	107.1	82.1	85.6	59.4	41.5	48.8	94.2	53.7
*P3*	921.8	1050.2	907.6	892.8	949.5	1266.5	1158.8	1003.1
*P4*	12.3	11.6	11.2	28.4	15.7	21.1	33.3	45.7
*P5*	36.0	40.1	33.5	86.9	66.7	28.6	57.1	72.6
*P6*	27.7	31	31.3	18.7	12.2	16.3	15.4	13.6
*Y*	197.3	175.2	166.6	155.9	163.7	169.5	176.7	173.9

#### 3.3.2 Results of gray correlation analysis

The results calculated according to Eq. 8 are shown in Table 3. The correlation between the peak areas of the six common peaks and the pharmacodynamic comprehensive evaluation values were all >0.6, indicating the presence of a certain correlation between the compounds corresponding to these six peaks and the inflammation indicators. Among these, the correlation degree between the *P2* peak and the pharmacodynamic comprehensive evaluation value was >0.8, suggesting that the correlation between the compound corresponding to the *P2* peak and the inflammation indicators are better. The correlation degree between the *P1* and *P3* peaks and the pharmacodynamic comprehensive evaluation value was >0.9, implying that the correlation between the compound corresponding to these two peaks and the inflammation indicators are in an excellent state.

**TABLE 3:** Gray correlation value between the ingredients and the comprehensive evaluation value of efficacy.

	*P1*	*P2*	*P3*	*P4*	*P5*	*P6*
*Y*	0.921	0.825	0.931	0.743	0.720	0.788

#### 3.3.3 Results of multiple linear regression analysis

The relationship between the pharmacodynamic comprehensive evaluation value and the area of each chromatographic peak was calculated using multiple linear regression analysis, as shown in Eq. 14.

Y=151.257−0.014P1+0.232P2+0.001P3+0.302P4−0.100P5+0.206P6 (14)

#### 3.3.4 Results of partial least-square analysis

After standardizing the data, the principal components of the two variable groups were extracted according to Eqs 10, 11, and two principal components were extracted from the independent variables. The relationship between the two principal components and each peak area (standardized data) was as follows:

$$t_{1}=-0.538P_{1}-0.411P_{2}-0.319P_{3}-0.184P_{4}-0.386P_{5}-0.508P_{6},$$

$$t_{2}=0.343P_{1}-0.585P_{2}+0.295P_{3}-0.070P_{4}+0.438P_{5}-0.510P_{6}.$$

According to Eq. 12, the regression coefficients between *Y* and the two principal components were −0.293 and −0.210, respectively.

The relationship between the pharmacodynamic comprehensive evaluation value (actual value) and the peak area of each chromatographic peak (actual value) was obtained according to Eq. 13. The result is shown in Eq. 15.

y=143.460+0.038P1+0.118P2+0.002P3+0.064P4+0.009P5+0.412P6 (15)

#### 3.3.5 Comparison of the two mathematical models

The predicted value of the pharmacodynamic comprehensive evaluation value *Y* was calculated using Eqs 14, 15. The results are shown in Table 4.

**TABLE 4:** The predicted value, actual value, and D-value of pharmacodynamic comprehensive evaluation value (Y).

*Y* (actual value)	*Y* (predicted value)^1^	Difference^1^	*Y* (predicted value)^2^	Difference^2^
197.252	184.314	12.938	182.135	15.117
175.222	176.053	4.931	177.225	6.356
166.639	176.720	10.081	176.683	10.044
155.926	167.122	11.196	170.749	14.823
163.653	159.542	8.839	162.883	7.880
169.499	167.385	5.137	167.613	4.416
176.693	178.419	8.342	173.402	10.756
173.881	169.208	13.555	168.073	12.628

Note: predicted value ^1^ was calculated by using a multiple linear regression model. The predicted value ^2^ was calculated by using a partial least squares regression model.

As shown in Table 4, the prediction results of the two mathematical models were similar. The total error between the predicted and actual values of the pharmacodynamic comprehensive evaluation value as per multiple linear regression was 225.057. However, the total error between the predicted and actual values of the pharmacodynamic comprehensive evaluation value according to the partial least-squares method was 246.059. Based on these results, it can be seen that the predicted value calculated using multiple linear regression was closer to the actual value. Therefore, this method was used in this experiment to establish the correlation between the peak areas of the six chromatographic peaks and the pharmacodynamic comprehensive evaluation value. The correlation coefficients between each chromatographic peak area and those between the peak area and the comprehensive evaluation value of pharmacodynamics in the medicated plasma were calculated. The results are shown in Table 5.

**TABLE 5:** The correlation coefficients between the individual chromatographic peak area and between the peak area and the comprehensive evaluation value of pharmacodynamics.

	*P1*	*P2*	*P3*	*P4*	*P5*	*P6*	*Y*
*P1*	1.000	0.345	0.504	−0.078	−0.341	0.239	0.221
*P2*	0.345	1.000	−0.187	−0.229	−0.375	0.636	0.556
*P3*	0.504	−0.187	1.000	0.005	−0.280	−0.208	−0.055
*P4*	−0.078	−0.229	0.005	1.000	0.637	−0.495	0.010
*P5*	−0.341	−0.375	−0.280	0.637	1.000	−0.659	−0.276
*P6*	0.239	0.636	−0.208	−0.495	−0.659	1.000	0.410
*Y*	0.221	0.556	−0.055	0.010	−0.276	0.410	1.000

Y represents the correlation coefficient between the peak area and the comprehensive evaluation value of pharmacodynamics.

As shown in Table 5, the correlation coefficients between the areas of the chromatographic peaks were all <0.7. The correlations between the ingredients were small, which signified that most of the ingredients were independent of each other. According to the absolute value of the correlation coefficient, the correlation between the chemical ingredients represented by the chromatographic peak and the pharmacodynamic comprehensive evaluation values was ranked as follows: *P2* > *P6* > *P5* > *P1* > *P3* >*P4*.

## 4 Discussion

TCM compounds are composed of two or more types of TCMs, and their efficacy is closely related to their chemical composition. However, the chemical components in TCM compounds are quite complex, with multiple components acting synergistically, and their pharmacological effects being chiefly exerted on the blood components (Wang et al., 2017). Therefore, it is extremely important to study the relationship between the chemical components of TCMs and their efficacy as well as to examine the prototype components and metabolites in the plasma samples. For example, past studies have demonstrated that the “Qi-invigorating” and “blood-activating” components in Qishen Yiqi Fang can confer protection against cerebral ischemia injury by regulating the inflammatory response and autophagy (Wang et al., 2022). Moreover, the spectrum-effect correlation can be applied to determine and describe the intrinsic relationship between the chemical constituents in TCM compounds and their efficacy (Luo et al., 2019; Zhang et al., 2019). Therefore, this study attempted to identify the chemical constituents in the YYTNG plasma samples that were highly correlated with inflammatory indicators by establishing the spectral-effect correlation between the HPLC fingerprint of YYTNG in MCAO rat plasma samples and inflammatory indicators.

TNF-α is the earliest and the most important inflammatory response marker (Dinarello, 2000) that can activate neutrophils and lymphocytes and enhance cell permeability (Cattani-Cavalieri et al., 2019). Excess TNF-α could promote the synthesis and the release of other inflammatory factors as well as aggravate the inflammatory injury. IL-18 is an important inflammatory factor in the IL-1 family, which is predominantly secreted by epithelial cells, dendritic cells, and macrophages (Dinarello, 2002). IL-18 is the main cytokine downstream of NLRP3 inflammasome that can be released by inflammasome at the time of inflammation (Zhang et al., 2021). As the downstream indicators of the JAK2/STAT3 signaling pathway (Raible et al., 2014; Qin et al., 2019; Sun et al., 2020), IL-18 and TNF-α are the key factors in the inflammatory response (Zhou et al., 2016; Li et al., 2018). Previous research has shown that YYTNG could significantly improve the inflammatory reaction in MCAO rats by reducing the levels of IL-18 and TNF-α in the plasma. The exogenous rCX3CL1 could significantly inhibit the activation of NLRP3 and NF-κB and reduce the expression of IL-1β and IL-18 (Ge et al., 2022). The death-associated protein kinase (DAPK) family is one of the important families of serine/threonine kinases involved in regulating certain biological functions in human cells. Past studies have demonstrated the inhibition of TNF-α and IL-1β expression via modulation of DAPK1 signaling in stroke (Noori et al., 2022). Therefore, TNF-α and IL-18 were selected in this experiment to explore the spectrum-effect relationship of YYTNG in MCAO rats. The results of our research revealed that the levels of inflammatory factors in the plasma of rats decreased with time. Thus, YYTNG can reduce the levels of related inflammatory factors in the plasma of MCAO rats, thereby significantly alleviating the inflammatory injury. The findings indicated that the levels of inflammatory factors decreased with time, implying that YYTNG could reduce the levels of the related inflammatory factors in the plasma of MCAO rats, thereby significantly improving the inflammatory injury.

Mathematical models are often used to evaluate the relationship between the fingerprints of TCM compounds and their efficacy as well as to determine the chromatographic peaks closely related to pharmacodynamics from the fingerprints (Feng et al., 2020; Bai et al., 2021). We had previously investigated the *in vitro* spectrum-activity relationship of YYTNG (Yu et al., 2021). However, the *in vitro* chemical fingerprints could not accurately assess the relationship between TCM components and their efficacy (Dan-dan Wang et al., 2014; Wei et al., 2018). Medicated plasma is more consistent with what is actually happening within the human body (Lin et al., 2016) as it can retain more TCM components (He et al., 2005). Therefore, the medicated plasma was selected for determination via HPLC in this experiment. Six common peaks with good resolution (*R* > 1.5) and high peak area were detected in the HPLC fingerprints of the YYTNG plasma samples. The results demonstrated that the peak area of *P3* was large, which indicated the presence of a correspondingly high amount of chemical ingredients in the medicated plasma. The chemical ingredients in the plasma changed with time, and the areas of most of the peaks decreased with time; however, the peak areas increased after 24 h owing to repeated oral administration. Although HPLC can determine the common peak areas in the plasma, it cannot discern the chemical structure of the ingredients represented by these peaks. Therefore, this experiment provides a strong basis for further investigations on the changes in the constituents of YYTNG in the plasma of MCAO rats.

Different mathematical models could be used to obtain varying results. Therefore, this study established the spectrum-effect relationship between YYTNG fingerprints and inflammatory factors in the plasma of MCAO rats via three mathematical models, namely gray correlation, multiple linear regression, and partial least squares. To eliminate possible multicollinearity among multiple independent variables (Franke, 2010), common peaks in the *in vivo* drug-containing plasma were denoted as P1-P6. Y represents the comprehensive evaluation value of the plasma pharmacodynamics in the MCAO rats. The results of gray correlation analysis showed that the correlations between the six chromatographic peaks and the comprehensive evaluation value of pharmacodynamics were all >0.6, thus signifying that the six components in the YYTNG fingerprint had a certain correlation with the inflammatory indicators. SPSS Statistics 25 software and Matlab R2018b software were used to select the six chromatographic peaks for correlation analysis using multiple linear regression and the partial least-square method. The comprehensive evaluation value of pharmacodynamics was predicted using a regression equation. The results indicated that the predicted values were not very different from the actual values. Furthermore, the error between the predicted value and the actual value calculated using multiple linear regression was small. Therefore, the multiple linear regression analysis was employed to analyze the correlation between the six chromatographic peaks and the comprehensive evaluation value of pharmacodynamics. As shown in Table 5, the absolute correlation coefficients between the peak areas of *P2*, *P6*, *P5*, and *P1* and the comprehensive evaluation value of pharmacodynamics were all ≥0.2. These results alluded that the chemical constituents corresponding to these peaks were significantly associated with the inflammatory factors. Although no highly correlated chromatographic peaks were identified in this experiment, these compounds can be isolated and analyzed in the future.

This experiment established the spectrum-effect relationship between the HPLC fingerprint of YYTNG in the plasma of MCAO rats and the inflammatory indicators. The present findings have laid the foundation for the further determination of the main anti-inflammatory injury components of YYTNG in MCAO rats. The limitation of this study is that it failed to identify several chromatographic peaks with a high correlation. In future research, we intend to identify these chromatographic peaks with a high anti-inflammatory correlation to analyze the YYTNG plasma prototype components and the metabolite components related to anti-inflammatory injury in plasma samples.

## 5 Conclusion

In this experiment, mathematical models were used to analyze the correlation between HPLC fingerprints and inflammatory indicators in MCAO rats. The results revealed a relatively large correlation between six chromatographic peaks in rat plasma and inflammatory indicators, albeit the results were inadequate. This could be because the six chromatographic peaks have not been identified. Therefore, in subsequent experiments, we plan to combine various experimental techniques to isolate and identify these six components and further verify the relationship between them and inflammatory indicators through cell experiments. This study lays a preliminary experimental foundation for the separation and structural identification of the chemical components represented by the six common peaks in the later period, thereby providing an idea for the establishment of *in vivo* fingerprints of traditional Chinese medicine compounds and drug metabolism research. The combination of mathematical models and *in vitro* and *in vivo* experiments is expected to provide certain guiding significance for clinical rational drug use and safety evaluation.

## Data Availability

The original contributions presented in the study are included in the article/Supplementary Material, further inquiries can be directed to the corresponding authors.

## Abbreviations

CCA, Common Carotid Artery; ECA, External Carotid Artery; ELISA, Enzyme Linked Immunosorbent Assay; HPLC, High-Performance Liquid Chromatography; IL, Interleukin; ICA, Internal Carotid Artery; MCAO, Middle Cerebral Artery Occlusion; OD, Optical Density; SD, Sprague Dawley; SPF, Specific Pathogen Free; TCM, Traditional Chinese Medicine; TNF, Tumor Necrosis Factor; YYTNG, Yangyin Tongnao Granules.
